# A Propensity-Score Matching Comparison between 27-Gauge and 25-Gauge Vitrectomy Systems for the Repair of Primary Rhegmatogenous Retinal Detachment

**DOI:** 10.1155/2019/3120960

**Published:** 2019-01-10

**Authors:** Daniele Veritti, Valentina Sarao, Paolo Lanzetta

**Affiliations:** ^1^Department of Medicine-Ophthalmology, University of Udine, Udine, Italy; ^2^Istituto Europeo di Microchirurgia Oculare (IEMO), Udine, Italy

## Abstract

**Purpose:**

To compare the anatomical and visual results and complications of 27-gauge versus 25-gauge transconjunctival sutureless vitrectomy for the management of primary rhegmatogenous retinal detachment.

**Methods:**

A prospective, propensity score-matched 6-month study was performed. All patients underwent either 27-gauge or 25-gauge vitrectomy as the first surgical intervention and were followed up over a 6-month period, in order to evaluate anatomical success, change in best-corrected visual acuity (BCVA), and intraoperative and postoperative complications including intraocular pressure dysregulation.

**Results:**

Propensity score matching resulted in two groups of 37 eyes each. All eyes completed a six-month follow-up. Baseline demographic and preoperative ocular characteristics showed no statistically significant difference between the two cohorts. The single operation success rate was 33/37 (89%) for 27-gauge cases and 34/37 (92%) for 25-gauge cases (*p*=0.7). The final anatomical success rate was 100% for each of the two cohorts. Mean BCVA change at the 6-month postoperative follow-up visit was −0.67 logMAR in the 27-gauge group and −0.71 logMAR in the 25-gauge group (*p*=0.9). Two patients in the 25-gauge group experienced transient hypotony after surgery.

**Conclusion:**

No significant difference between 27-gauge and 25-gauge transconjunctival sutureless vitrectomy for the repair of primary rhegmatogenous retinal detachment was recorded in terms of reattachment rate, BCVA, intraoperative and postoperative complications.

## 1. Introduction

Rhegmatogenous retinal detachment (RRD) is defined as the separation of the neurosensory retina from the retinal pigment epithelium layer secondary to the presence of retinal breaks, allowing the accumulation of fluid in the subretinal space.

Over the past decades, the management of RRD has been revolutionized. Rhegmatogenous retinal detachment has changed from an untreatable condition leading to permanent vision loss to a repairable event after which visual improvement is very likely. A range of surgical techniques have been employed over the years to manage this sight-threatening pathology, including pneumatic retinopexy, scleral buckling, and pars plana vitrectomy (PPV) [1, 2]. Pars plana vitrectomy is nowadays the most commonly used procedure to repair primary RRD [3–5]. In the recent past, it has been progressively refined, thanks to technological progress, such as the development of small-gauge instrumentation and the use of high-speed probes. The main force driving PPV technical advancement is making a successful procedure less invasive, safer, with quicker recovery, and possibly improved outcomes. Indeed, smaller sutureless sclerotomy wounds result in less postoperative inflammation, improved patient comfort, and faster recovery.

Transconjunctival sutureless 27-gauge PPV has emerged as an important advancement in vitreoretinal surgery instrumentation [6, 7]. At the beginning, it was employed in noncomplex cases, such as vitreous hemorrhage and posterior pole procedures [7, 8]. More recently, surgeons advocate its use in more complicated conditions, including RRD requiring silicone oil tamponade [9–15].

Besides being less invasive when compared to 25-gauge PPV, 27-gauge PPV carries additional potential advantages, thanks to the design of the vitrectomy probe. It generates the shortest attraction distance and a smaller “sphere of influence” compared with 23-gauge and 25-gauge vitrectomy systems. This allows a more accurate fluid control and a greater dissection precision, theoretically allowing for safer procedures with reduced iatrogenic breaks [16].

However, 27-gauge vitrectomy also carries potential drawbacks when compared to 23-gauge and 25-gauge systems for the treatment of RRD. These limitations include a reduction in the flow rate potentially influencing the efficiency of the procedure, an increased instrument flexibility especially during anterior maneuvers, and the potential underfilling of tamponade [7, 17].

Evidence comparing 27-gauge to 25-gauge PPV for RRD is still limited [9–12]. Therefore, we designed and conducted the present study to compare the efficacy (in terms of reattachment rates after single or multiple surgeries) along with the safety of new high-speed 27-gauge PPV versus 25-gauge PPV for the repair of primary RRD in phakic and pseudophakic eyes.

## 2. Materials and Methods

### 2.1. Study Design

This is a 6-month, prospective, single-center, comparative, propensity score-matched study. It follows the tenets of the Declaration of Helsinki. Data were prospectively collected at the Department of Medicine-Ophthalmology, University of Udine, Udine, Italy. Patients with primary RRD were included in this study. Exclusion criteria were as follows: (1) inability to give informed consent; (2) previous ocular surgery excluding noncomplicated cataract extraction; (3) history of penetrating ocular trauma; (4) significant ocular comorbidities, such as uveitis, uncontrolled glaucoma, proliferative diabetic retinopathy, and proliferative vitreoretinopathy (PVR) greater than grade C accordingly to the updated Retina Society Classification [18]; (5) severe systemic disease.

Patients underwent either 27-gauge PPV or 25-gauge PPV, and we adopted a propensity score matching strategy to correct the selection biases and to compare the two groups. Propensity score method used a multivariable logistic regression model based on the following preoperative characteristics: age, macula status (on, off), lens status, presence and grade of PVR, number of retinal breaks, and best-corrected visual acuity (BCVA). Using predicted probabilities, we matched a participant in the 27-gauge group with the closest individual in the 25-gauge group using the nearest-neighbour matching technique.

### 2.2. Examinations

Preoperative evaluation consisted of a complete medical, surgical, and ophthalmic history followed by a thorough ophthalmic examination. Best-corrected visual acuity was measured using ETDRS charts and reported as logarithm of the minimum angle of resolution (logMAR). The updated Retina Society Classification was used to grade PVR, if present [18]. Patients were examined at baseline and then at days 1, 7, and 15 and at months 1, 3, and 6 after surgery.

### 2.3. Surgical Technique

All PPVs were performed under local anesthesia with a retrobulbar block. All surgeries were performed by the same surgeon (PL). Concurrent phacoemulsification and intraocular lens implantation were performed before vitrectomy if cataract was present. Surgical procedures were performed using the Alcon Constellation (Alcon, Forth Worth, TX, USA) under a Resight 700 (Carl Zeiss Meditec AG, Oberkochen, Germany) noncontact panoramic viewing system. Both the 27-gauge and 25-gauge procedures were performed using a three-port pars plana technique (the third port was used for a 25-gauge chandelier illuminator). Either 27-gauge or 25-gauge sclerotomies were created using the trocar cannula with a biplanar entry in order to create a self-sealing access. In detail, after displacement of the conjunctiva, oblique incisions at an angle of 45 degrees to the sclera with a two-step technique were performed.

The surgical parameters for both groups were the following: fixed cut rate of 7,500 cuts per minute (cpm), proportional vacuum of up to 650 mmHg, and intraocular pressure (IOP) at 35 mmHg. Posterior hyaloid detachment, core vitrectomy, and extensive peripheral vitrectomy over 360 degrees were performed in all cases. The vitreous base was meticulously shaved circumferentially in all cases with scleral depression. Any tears or suspicious retinal lesions were treated with endolaser photocoagulation or trans-scleral cryopexy. Perfluorocarbon liquid (PFCL) was used intraoperatively at surgeon discretion. After air-fluid exchange, 12% perfluoropropane (C3F8) gas, or 1,000 centistoke silicone oil was used as tamponade. At the end of the procedure, microcannulas were removed and a gentle massage of the sclerotomy with a cotton-tipped applicator was performed to avoid leakage; if any site showed persistent leakage, 7-0 vicryl sutures were placed.

### 2.4. Outcome Measures

The primary outcome measure was the retinal reattachment rate at month 6 (after single or multiple procedures). Cases were judged successful if retina's reattachment was maintained without tamponade agents.

Secondary outcomes were anatomical success at month 6 after a single procedure, operating time, rate of silicone oil utilization, BCVA at month 6, IOP dysregulation (hypotony/hypertony), and intraoperative and postoperative complications.

### 2.5. Statistical Analysis

Nonparametric tests (Wilcoxon test) and parametric tests (two-tailed *t*-test) were used to assess non-normally and normally distributed data, respectively. Dichotomous measures were compared using a chi-square test and Fisher's exact test. A *p* value of < 0.05 was defined as statistically significant.

## 3. Results

### 3.1. Subject Characteristics

The comprehensive pool of patients used to create the propensity score matching consisted of 114 eyes (41 in the 27-gauge group and 73 in the 25-gauge group). One-to-one matching according to the propensity score resulted in the two groups containing 37 eyes each. Baseline characteristics are listed in Table 1. As expected, due to the propensity score matching method, there was no preoperative statistically significant difference for each parameter between the two groups. All patients completed the 6-month follow-up.

### 3.2. Vitrectomy Outcomes

Retinal breaks could be identified in 100% of cases intraoperatively in both groups. Instrument sclerotomies were sutured in 3 cases (8%) in the 27-gauge group and in 11 cases (29%) in the 25-gauge group (*p*=0.017). Twenty-five-gauge access for illuminated chandelier was excluded from analysis.

Operative time was comparable between the two groups (27-gauge: 86.1 ± 30.1 minutes; 25-gauge: 90.7 ± 29.6 minutes; *p*=0.5). Perfluorocarbon liquid was given in all cases. In the 27-gauge group, 28 eyes (76%) had C3F8 gas tamponade and 9 eyes (24%) needed silicone oil. In the 25-gauge group, 30 eyes (81%) had C3F8 gas tamponade and 7 eyes (19%) had silicone oil tamponade (Table 2). The use of silicone oil was at the surgeon's discretion. It was used in cases with multiple, large, inferior breaks. Patients who received silicone oil tamponade underwent a second surgical procedure to remove the oil within 4 months from the initial surgery. At the final visit, no patient had silicone oil tamponade.

### 3.3. Anatomical Results

Anatomical success after a single operation at month 6 was 89% and 92% in the 27-gauge and in the 25-gauge groups, respectively. The difference was not statistically significant (*p*=0.7). Recurrence of retinal detachment occurred in 4 eyes operated with a 27-gauge system and in 3 eyes in the 25-gauge group. All redetachments occurred within 3 months from the initial surgery. All patients presented with an attached retina at the 6-month follow-up visit (Table 2).

### 3.4. Visual Acuity Results

**Figure 1:**
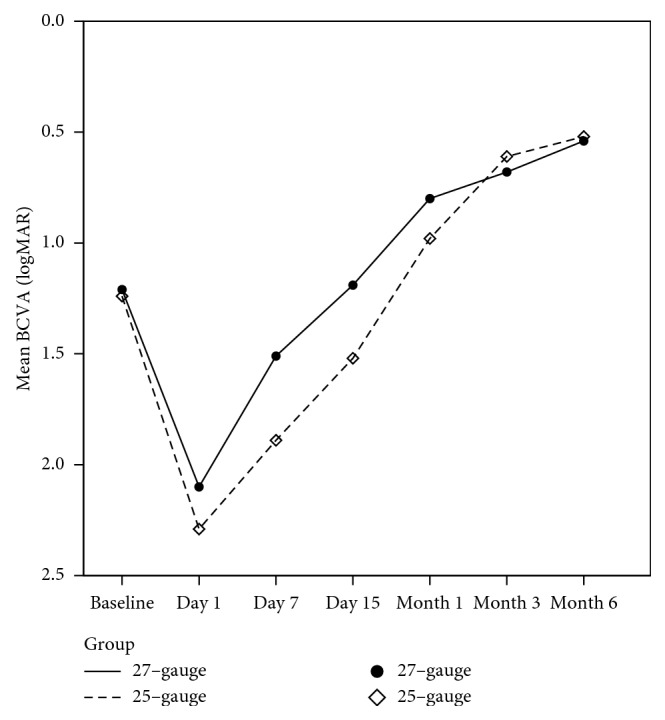
Best-corrected visual acuity changes.

Best-corrected visual acuity changes are summarized in Figure 1. After the surgery, mean BCVA significantly improved in both groups (*p*=0.006 and *p*=0.004 in the 27-gauge and 25-gauge groups, respectively). Mean BCVA change at the 6-month postoperative follow-up visit was −0.67 logMAR in the 27-gauge group and −0.71 logMAR in the 25-gauge group (*p*=0.9) (Table 2). At the 6-month visit, BCVA improved by more than 1 ETDRS line in 32 cases (86%) in the 25-gauge group and in 33 eyes (89%) in the 27-gauge group (*p*=0.7). Vision was stable in the remaining cases. No visual acuity decrease of more than 5 ETDRS letters was recorded at the end of the follow-up.

### 3.5. Complications

No severe intraoperative complications occurred among the 74 eyes included in this analysis. The surgeon did not need to change the chosen instrumentation in any case. None of the eyes experienced significant intraocular inflammation after surgery. At day 1, no substantial differences in the amount of tamponade agents were noted in the two groups: a gas filling ≥90% was recorded in all (100%) eyes in the 27-gauge group and in 28 (93%) eyes in the 25-gauge group (*p*=0.6). None of the patients in the 27-gauge group presented with IOP inferior to 10 mmHg on Goldmann applanation tonometry. Conversely, 2 cases experienced transient hypotony (IOP < 10 mmHg) in the 25-gauge group. Severe hypertension (IOP > 30 mmHg) was detected in 4 eyes (11%) in the 27-gauge group and in 5 eyes (14%) in the 25-gauge group during the follow-up (Table 2). In all cases, the raised IOP was transient and successfully treated with topical medications. No other postoperative complication was noted in the follow-up period in either group.

## 4. Discussion

The surgical management of RRD has come a long way over the past decades. Significant steps ahead have been made, and a variety of techniques are now available [19]. Recent years have witnessed a gradual increase in the application of small-gauge sutureless vitrectomy, which has now become a primary treatment modality in the management of RRD [3–5].

Recently, 27-gauge vitrectomy has been proposed for treatment of RRD. The main theoretical advantages of this technique are great precision of dissection maneuvers, effective fluidics, reduced postoperative inflammation and astigmatism, and improved wound integrity. However, concerns still exist and are related to operation efficiency, flexibility of instrumentation, and the possibility of wound leaks [9–14].

There are limited published data comparing the efficacy and safety of 27-gauge and 25-gauge PPV for RRD. Romano et al. [9] reported that the safety and efficacy of 27-gauge PPV for RRD appear similar to 25-gauge PPV. In both groups, anatomical success was obtained after a single round of surgery in 14 out of 15 eyes (93%). In another comparative study conducted by Rizzo et al. [10] on 40 eyes, the primary anatomical success rate after a single operation was 90.0% and 85.0% in the 27-gauge and in the 25-gauge groups (*p*=0.63), respectively. Otsuka et al. recently published a retrospective study on comparing 25-gauge (32 eyes) and 27-gauge (30 eyes) PPV for primary RRD, finding no significant anatomical or functional differences with a primary success rate of 96.7% (25-gauge) and 93.8% (27-gauge) [12].

In this comparative, propensity score-matched study, we observed a comparable single-procedure success rate of 89% for the 27-gauge vitrectomy group and 92% for the 25-gauge group (no statistically significant difference). All retinas were flattened after an additional vitrectomy surgery at month 6.

The main benefit we noticed using 27-gauge systems is the increased precision of the surgical maneuvers. Dugel et al. [16] reported that smaller-gauge instruments limit involvement of surrounding tissue during delicate membrane dissection, thanks to shorter membrane attraction distances and reduced “sphere of influence”. In addition, the 27-gauge vitrectomy probe used in this series features a port placed 0.2 mm from the tip. Having a port so close to the edge of the probe allows greater precision and fewer tractional manoeuvres [20]. In our comparative study, we recorded no cases of iatrogenic tears in either group, and both 27-gauge and 25-gauge instrumentation were adequate in obtaining precise dissection also in the cases complicated by PVR. An adequate membrane peeling was achieved with both 25-gauge and 27-gauge forceps. It is important to note that, with its smaller diameter, the 27-gauge cutter can more easily be placed in the space between the retina and the PVR membrane, which is helpful in membrane dissection [21].

Operation efficiency is one of the major theoretical concerns regarding 27-gauge instrumentation. The inner lumen radius of a 27-gauge probe is reduced by approximately 20% when compared to a 25-gauge probe. According to the Hagen-Poiseuille law, this should theoretically result in a decrease in the flow rate by approximately 60%. However, dual pneumatically operated vitrectomy probes with ultrahigh cut rates (7500 cpm) can maintain an efficient vitreous flow rate [21]. High cut rate lowers vitreous viscosity, resulting in reduced resistance to flow. Moreover, the 27-gauge probe has excellent fluidics, and it is very effective in shaving vitreous from areas of detached retina. For these reasons, the reduced diameter of the 27-gauge instrumentation does not significantly prolong the operating times, although the posterior vitreous detachment inducement is more laborious with 27-gauge instrumentation due to lower suction capacity. In our study, we recorded a 5-minute difference favouring the 27-gauge group. No statistically significant difference in operating times was found in other comparative studies [9–12].

The decreased diameter of the instruments also leads to undesired flexibility of instrumentation and difficulty with access to the far peripheral vitreous. Such flexibility may theoretically limit the extent of peripheral vitrectomy and may permit anterior vitreous to contract leading to postoperative retinal breaks. Similar to previous reports, our study found 27-gauge instrumentation to be of adequate stiffness to complete all surgical manoeuvres requested by RRD repair with similar rigidity and functionality to 25-gauge instrumentation [9–12].

Improved wound construction and integrity has been cited as a primary advantage for use of 27-gauge over 25-gauge systems. Our results support this presumption. In our comparative study, 11 eyes in the 25-gauge group required suturing of sclerotomies compared to 3 eyes in the 27-gauge group (*p*=0.017). Two cases in the 25-gauge group showed transient postoperative hypotony on day 1, while no cases of hypotony were recorded in the 27-gauge group.

In our study, we utilized two 27-gauge accesses for surgical instrumentation and one 25-gauge access for chandelier illuminator. This approach allows a complete surgical procedure without the need of assistance. This procedure has been previously reported in a 25-gauge versus 27-gauge comparative study for RRD [10]. However, it must be taken into account that this may limit the significance of certain comparisons, such as IOP outcomes and hypotony rates. Moreover, the results we observed using a 25-gauge chandelier may not be directly generalized and compared to procedures using a 3-port PPV with a light pipe.

There are limitations to our study that should be considered, such as the small number of eyes and the lack of randomization. However, the present study has several strengths, including its prospective design, the propensity score matching method, and the standardized, single-surgeon procedure. It is known that propensity score matching has some weakness. In particular, this approach cannot reach the level of evidence of randomized, controlled, masked clinical trials, mostly due to the effect of hidden bias due to dormant, unobserved confounders. Differently from randomization, a propensity score method can only ensure balance in measured, not unmeasured, covariables. Nevertheless, it is worthwhile to mention that this is the largest prospective comparative study regarding 27-gauge PPV in RRD and the two studied groups are well balanced and comparable. Our results show that 27-gauge PPV seems to be as safe and effective as 25-gauge PPV in RRD surgery. The primary success rate (single-surgery) and final anatomical success at month 6 showed no statistically significant difference between the 2 groups. No severe intraoperative complications were observed and, overall, postoperative complications were limited in both groups.

For these reasons, we believe that 27-gauge PPV, combined with a wide-angle viewing system and precise intraoperative localization and treatment of retinal breaks, provides excellent results as 25-gauge PPV, and therefore, it represents a valid treatment option for RRD. Further larger and randomized studies are required in order to definitely state if 27-gauge PPV is as safe and effective as 25-gauge PPV for RRD.

## Figures and Tables

**Table 1:** Baseline characteristics.

	25-gauge PPV	27-gauge PPV	*p* value
Number of patients	37	37	
Age, mean ± SD (years)	63.1 ± 12.5	63.9 ± 13.5	0.8
Male sex, *n* (%)	24 (64%)	26 (70%)	0.6
Right eye, *n* (%)	18 (49%)	23 (62%)	0.2
Phakic, *n* (%)	19 (51%)	20 (54%)	0.8
Macula on, *n* (%)	17 (46%)	17 (46%)	1
Number of breaks, mean ± SD	2.2 ± 1.2	2.4 ± 1.5	0.6
Number of inferior breaks, mean ± SD	1.01 ± 1	1.12 ± 0.91	0.8
Number of detached quadrants, mean ± SD	2.3 ± 0.6	2.2 ± 0.5	0.4
PVR B, *n* (%)	3 (8%)	3 (8%)	1
PVR C, *n* (%)	1 (3%)	1 (3%)	1
BCVA, mean ± SD, logMAR	1.24 ± 1.04	1.21 ± 1.04	0.9

SD: standard deviation; BCVA: best-corrected visual acuity; logMAR: logarithm of the minimum angle of resolution; *n*: number; PVR: proliferative vitreoretinopathy.

**Table 2:** Outcomes.

	25-gauge PPV	27-gauge PPV	*p* value
*N*	37	37	
Surgical time, mean ± SD (minutes)	90.7 ± 29.6	86.1 ± 30.1	0.5
Anatomical success at month 6, *n* (%)	37 (100%)	37 (100%)	1
Anatomical success at month 6 after a single operation, *n* (%)	34 (92%)	33 (89%)	0.7
BCVA change at month 6, mean ± SD, logMAR	−0.71 ± 0.96	−0.67 ± 1.01	0.9
C3F8 gas tamponade, *n* (%)	30 (81%)	28 (76%)	0.6
Silicone oil tamponade, *n* (%)	7 (19%)	9 (24%)	0.6
Sutured sclerotomies, *n* (%)	11 (29%)	3 (8%)	0.017
Hypotony (IOP < 10 mmHg), *n* (%)	2 (5%)	0 (0%)	0.5
Severe IOP increase (>30 mmHg), *n* (%)	5 (14%)	4 (11%)	0.7

SD: standard deviation; BCVA: best-corrected visual acuity; C3F8: perfluoropropane; IOP: intraocular pressure; logMAR: logarithm of the minimum angle of resolution; *n*: number; PPV: pars plana vitrectomy.

## Data Availability

The data used to support the findings of this study are available from the corresponding author upon request.
